# Predicting where Small Molecules Bind at Protein-Protein Interfaces

**DOI:** 10.1371/journal.pone.0058583

**Published:** 2013-03-07

**Authors:** Peter Walter, Jennifer Metzger, Christoph Thiel, Volkhard Helms

**Affiliations:** Center for Bioinformatics, Saarland University, Saarbrücken, Germany; Koc University, Turkey

## Abstract

Small molecules that bind at protein-protein interfaces may either block or stabilize protein-protein interactions in cells. Thus, some of these binding interfaces may turn into prospective targets for drug design. Here, we collected 175 pairs of protein-protein (PP) complexes and protein-ligand (PL) complexes with known three-dimensional structures for which (1) one protein from the PP complex shares at least 40% sequence identity with the protein from the PL complex, and (2) the interface regions of these proteins overlap at least partially with each other. We found that those residues of the interfaces that may bind the other protein as well as the small molecule are evolutionary more conserved on average, have a higher tendency of being located in pockets and expose a smaller fraction of their surface area to the solvent than the remaining protein-protein interface region. Based on these findings we derived a statistical classifier that predicts patches at binding interfaces that have a higher tendency to bind small molecules. We applied this new prediction method to more than 10 000 interfaces from the protein data bank. For several complexes related to apoptosis the predicted binding patches were in direct contact to co-crystallized small molecules.

## Introduction

Protein-protein interactions play important roles in most cellular processes [1], [2]. In the yeast *S. cerevisiae*, for example, interaction partners have been reported for more than 5 000 of the 6 000 yeast proteins [3]. In human cells, protein interactions are involved, among others, in signaling processes, such as in the MAPK cascade, and in regulatory processes, such as the G-protein activated processes of hormone detection. Therefore, protein interactions are of vital interest for pharmaceutical intervention. Currently, the main approach for designing inhibitors and modulators of protein-protein interactions aims at designing peptidomimetics that compete with the natural partner protein for the same interface [4]. As some of these binding interfaces can also bind small molecule ligands, modulating the activities of protein-protein complexes by competitive or allosteric small molecule protein-protein inhibitors (SMPPIs) has become an area of very active interest in current pharmaceutical research [5], [6]. Although rational design of SMPPIs still presents a considerable challenge [5], promising progress has been made in several cases towards finding small molecules that efficiently inhibit protein-protein interactions. A prominent example is the p53-MDM2 system that is a promising putative target for cancer therapy [7].

Our structural understanding of how proteins interact with other proteins and with small molecule ligands is largely based on the atomistic three-dimensional protein structures deposited in the Protein Data Bank (PDB) [8]. Statistical analysis of these complexes has allowed deriving some general principles about the binding interfaces of protein complexes. For example, permanent complexes tend to have large and hydrophobic interfaces whereas transient interactions often involve binding via smaller and more polar interfaces [9]. Besides, some binding interfaces resemble an O-ring where a hydrophobic interior is surrounded by a ring formed of polar and charged residues [10]. Protein binding interfaces are rather flat, on average, particularly when compared to those involved in binding small ligands that often bind into pronounced clefts on the protein surface. Yet, binding of ligands and/or the natural conformational dynamic fluctuations of proteins may induce the formation of binding pockets of suitable size and polarity as shown for several systems such as IL2-IL2-R, p53-MDM2, and Bcl-XL [5], [11].

Interestingly, not all interface residues play the same role for the stability (binding affinity) of the complex. There often exists a subset of interface residues, the so-called hot spots, that are mainly responsible for the binding affinity [10], [12] and may be promising locations for binding of small molecules. Moreover, these hot spot residues are generally not spread over the entire interface but are located in clusters [13], [14]. Thus, one could expect that successful SMPPIs preferentially bind in regions where hot spots residues are enriched. Besides experimental information on the location of hot spots, several fast computational prediction-algorithms are available for predicting hot spot residues at protein-protein interfaces [15], [16] with about 70% accuracy [17]. Kozakov et al. have recently demonstrated for 15 PPI target proteins that druggable sites comprise a cluster of binding hot spots with rather concave topology[18]. In general, the binding pockets were formed by a mosaic-like pattern of hydrophobic and polar amino acids so that they could accommodate well a mixture of 16 organic probe molecules in a procedure termed ‘computational solvent mapping’. Furthermore, Metz et al. selected promising configurations for ligand docking from an ensemble of protein conformations [19] based on predicted hot spot residues from MM-PBSA calculations.

Very few studies have compared the general properties of protein-protein and protein-ligand complexes [7], [20]–[22]. The Timbal database contains structural data for a small number of protein-protein complexes and their complementary protein-ligand inhibitor complexes [23]. Davis and Sali [24] compiled a large dataset of protein-protein and protein-ligand complexes. They classified surface residues at the binding interface into ‘bifunctional sites’ that contain residues that bind to other proteins as well as to small-molecule ligands, and ‘mono-functional sites’ that only interact with other proteins. Bifunctional sites can be predicted with high accuracy by a homology transfer algorithm termed HOMOLOBIND when homologous template binding sites are known [25]. Koes and Camacho predicted so-called ‘small-molecule inhibitor starting points’ (SMISPs) on the surface of the protein binding partner [26]. Their statistical classifier achieved 70% leave-one-complex-out cross-validation accuracy. Similar to these two works, we analyze here PP:PL pairs in which a ligand L_j_ and a second protein P_i2_ compete for the same binding interface on the surface of the first protein P_i1_/P_i3_. For these interfaces, our method predicts ‘where at the binding interface small-molecule ligands will bind most likely’.

## Materials and Methods

### Compilation of non-redundant dataset

All interface data and features were retrieved from our ABCsquare database [27] that is based on the structures of biomolecular protein-protein and protein-ligand complexes taken from the PDB database [8]. For each complex, the ABCsquare database provides a list of interface residues that were identified using a distance based approach. Any surface residue is considered as interface residue if at least one residue from the binding partner can be found within a radius of 5 Å.

The main dataset for this study (see table S1) contains a list of PP:PL pairs where one protein may bind either a second protein or a small molecule ligand at the same interface. At first, we compiled a non-redundant set of tuples (P_i1_, P_i2_)∶(P_i3_, L_j_) where P_i1_, P_i2_ and P_i3_ are three proteins and L_j_ is a small molecule ligand. Precisely, we considered small molecules that are defined as ‘HETATOMs’ in the PDB file except for ions and water molecules. Valid tuples were required to fulfil the following four conditions:

1. P_i1_ and P_i2_ are members of a protein-protein complex that is deposited in the PDB.
2. P_i3_ and L_j_ are members of a protein-ligand complex that is deposited in the PDB.
3. P_i1_ and P_i3_ share at least 40% sequence identity.
4. The aligned positions in the binding interfaces of P_i1_–P_i2_ and P_i3_–L_j_ have at least two residues in common.

**Figure 1:**
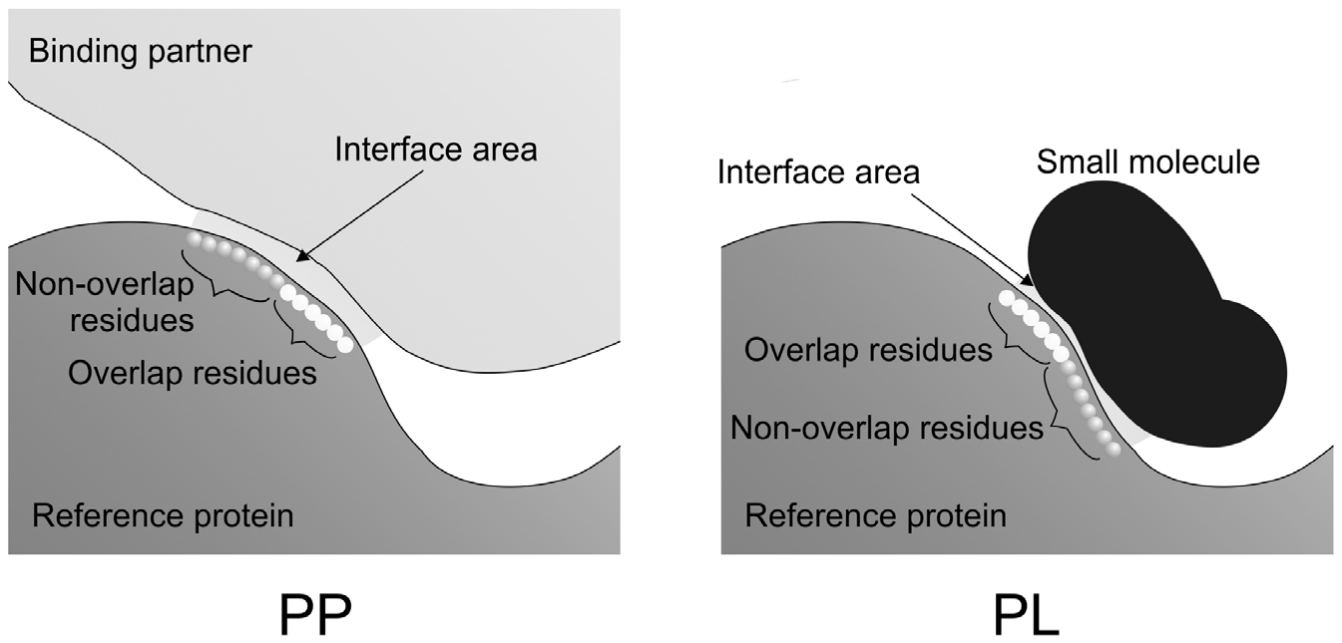
Schematic representation of a PP complex (left) and a PL complex (right). PP and PL share an identical or similar ‘reference protein’. The interface areas shown in light grey were determined using a distance criterion as described in the methods section. Overlap residues form contacts in the PP complex as well as in the PL complex whereas non-overlapping residues form contacts only in one of the complexes.

In the following we denote P_i1_ and P_i3_ as reference proteins as they determine the relation between the tuples (P_i1_, P_i2_) and (P_i3_, L_j_). For checking the last condition, the sequences of the reference proteins were aligned to each other. This resulted in a mapping of the respective interface regions. Residue pairs of proteins P_i1_ and P_i3_ that belong both to the P_i1_–P_i2_ as well as to the P_i3_–L_j_ interface were termed ‘overlapping’ residues. The remaining interface residues of P_i1_ were termed ‘non-overlapping’ residues, see Fig. 1. We did not consider PP:PL pairs that contained only one overlapping residue as this marginal overlap was considered being too small for deriving a meaningful statistical classifier. This procedure resulted in a dataset of about 10 000 pairs of complexes.

**Figure 2:**
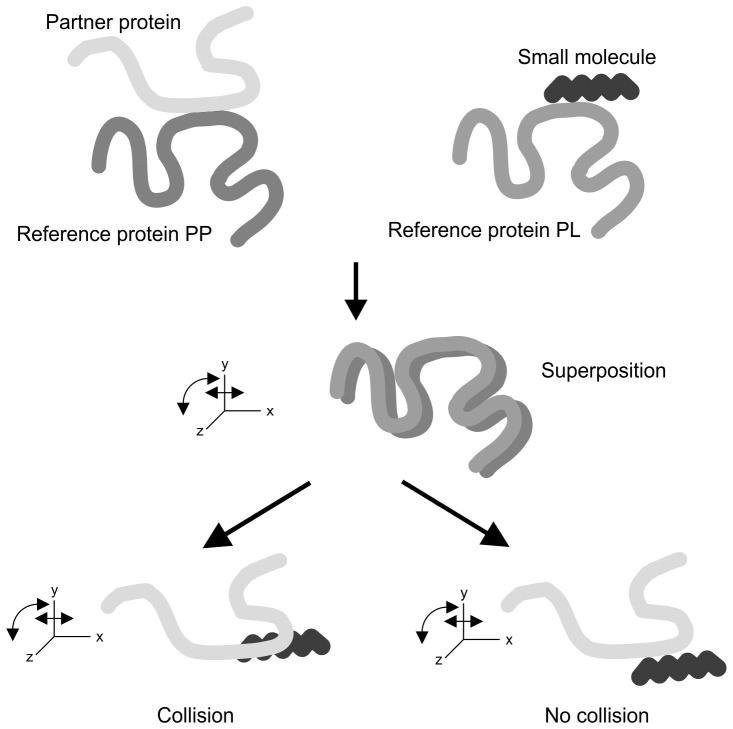
Identification of competitive PP:PL pairs using structural superposition.

However, this dataset may also contain PP:PL pairs where the ligand does not actually compete with the second protein for the interface on protein P_i1_, but both L_j_ and P_i2_ may bind simultaneously, possibly in a cooperative manner. As this work focuses on identifying competitive binders, the reference proteins P_i3_ were geometrically mapped onto the reference proteins P_i1_ using structural superposition using the program VMD [28], see Fig. 2. The resulting transformation and rotation matrices were then applied to the ligand L_j_. If any of the distances between the heavy atoms of L_j_ and P_i2_ was shorter than the sum of the two atomic radii this indicated a collision. In that case, L_j_ and P_i2_ are not likely to bind simultaneously to the same binding region on P_i1_. All other pairs of complexes were deleted from the list. This led to about 1 000 pairs of PP and PL interfaces in total.

In order to remove sequence redundancy among the PP:PL pairs we clustered the reference proteins P_i1_ of the remaining PP interfaces using the CD-hit program [29] with a sequence identity cut-off of 40%. This resulted in about 300 clusters. Every cluster contained one or several PP:PL pairs with homologous reference proteins. We did not want to consider short peptide fragments as representatives for the proteins P_i2_ as such peptides may only cover parts of the full PP interface. Therefore, we excluded a cluster entirely if it contained only small peptides shorter than 5 residues as P_i2_ structures. Another requirement was that the two interfaces of a PP:PL pair should be similar to each other. To this end, we calculated the sequence identity for the sequence stretches of corresponding interface residues consisting of a combination of overlapping and non-overlapping residues for the PP complex and the PL complex, respectively. Within each cluster we selected the representative with the highest identity of interface residues. The final dataset comprises 175 PP:PL tuples. These are listed in the supplementary material.

### Computation of interface features

For all PP complexes of the final dataset, we computed structural and sequence features of the interfaces that reflect the role of individual residues in the complex (all in all 5815 residues). (1) The evolutionary conservation score for a single residue was obtained from Consurf-DB that provides pre-calculated normalized conservation scores for all PDB structures [30]. These scores are based on multiple sequence alignments using PSI-BLAST and MUSCLE, respectively, and on calculating the evolutionary conservation of each amino acid position in the alignment using the Rate4Site algorithm. According to this score, well conserved sequence positions have negative scores and flexible ones have positive scores. (2) A measure for the energetic contribution of the residues at the binding interface was obtained from a hotspot prediction using an in-house implementation of two knowledge-based prediction algorithms [15], [16] in our ABCsquare database. Benchmarking this approach on a representative set of protein-protein interactions yielded a very similar accuracy (71%) as the webserver implementation by Keskin and co-workers [15] (70% for our test data set).

Besides, we computed several structural features of the binding interfaces that characterize their packing density and curvature. (3) A measure representing the level of burial or exposal of residues was quantified by the protrusion value. For this, we used the implementation from ref. 31 that calculates a protrusion value for the *i*th atom in a molecular structure as:



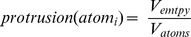



Here, *V_atoms_* denotes the volumes of the atoms within 10 Å radius around atom *i* and *V_empty_* represents the value of the remaining empty space in this sphere. An atomic protrusion value of 0 refers to fully buried atoms. The larger the value, the more exposed the atom is to the solvent. The protrusion value for an entire amino acid was computed as the average of the values over all atoms of this residue. Figure S1 in the supporting information shows an example of protrusion values that are visualized using different colors.

(4) The contact density of residue *i* was computed following Illingworth et al. [32] as the average contact density of its surface accessible heavy atoms according to:



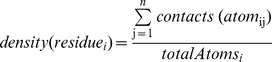



where *contacts(atom_ij_)* is the number of contacts between a surface accessible heavy atom *j* of residue *i* and other heavy atoms belonging to residues of the same chain within a radius of 5Å and *totalAtoms_i_* denotes the number of all heavy atoms in residue *i*.

(5) Also, for all interface residues *i* the relative surface fraction was calculated using the program Naccess [33]:



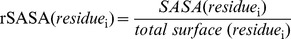



Here, the solvent accessible surface area (SASA) was calculated by Naccess for an individual residue *i* in a PP complex, whereby the total surface is the surface area of that residue located in the center of a tripeptide and surrounded by two alanines.

**Figure 3:**
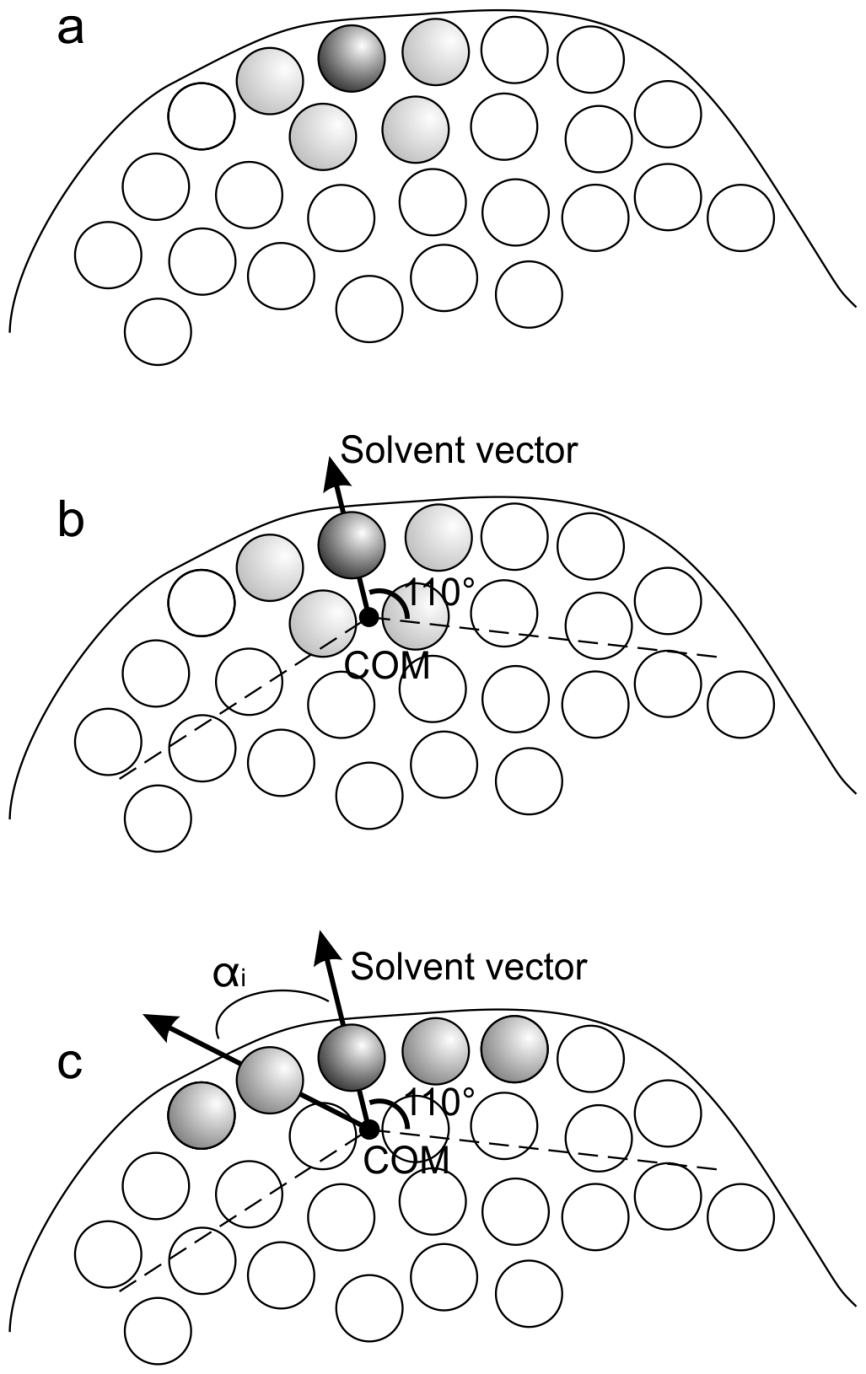
Construction of a surface patch. (a) Shown is a pre-patch of size *n = 5*, consisting of a central surface residue (dark grey) and *n-1 = 4* nearest neighbors residues (light grey). (b) Using the coordinates of the C_α_-atoms of the residues, the center of mass (COM) for this pre-patch is calculated. For any surface residue including the central residue, a solvent vector is defined using the coordinates of its C_α_-atom and the COM. (c) The final surface patch contains the central residue and the closest *n-1* surface residues (grey) for which the angle *α_i_* between their solvent vector and the solvent vector for the central residue is between 0° and 110°.

Next, we considered the direct neighbors of the residue of interest forming a small surface patch on the interface region. This approach was inspired by the work of Thornton et al. [34], [35]. A patch is made up of *n* surface residues, which consist of one central residue and *n-1* neighboring residues. Thus, a patch describes the microenvironment for a central residue with respect to geometric parameters or physico-chemical properties. We applied a reimplementation of the algorithm in ref. 34 and calculated patches for every surface residue in our dataset with sizes between 5 and 8. Fig. 3 outlines the construction of surface patches.

### Training of statistical classifier

The binary statistical classification of overlapping and non-overlapping residues was based on the random forests method [36] using the randomForest package from Breiman and Cutler implemented in R [37]. A random forest is a fast classifier consisting of a collection of decision trees, where each tree classifies a residue separately. The idea of such an ensemble classifier is to combine a number of weak learners to create a single strong learner. To obtain a single prediction, a majority vote is performed at the end. Each tree is trained using a different bootstrap sample from the original dataset (which is obtained by random sampling with replacement). For each node of a tree a subset of the available features is randomly selected and the best split on these is chosen according to the training set by using the Gini impurity criterion. The Gini impurity is a measure of how often a randomly chosen residue from the training set would be incorrectly classified if it were randomly classified according to the distribution of the two classes in the subset. Each tree is fully grown and not pruned. Because of the bootstrap sampling, about one-third of the original cases are left out of the training set of a specific tree and thus, they are not used in the construction of that tree. This data is used to get a running unbiased estimate of the classification error as trees are added to the forest. It is also used to obtain estimates for the importance of individual features. Because of that, there is no need for cross-validation or a separate test set to obtain an unbiased estimate of the test set error in random forests. In our study the random forests were trained with the five most promising features identified during this work (see above). The default parameters were employed for the number of trees and the number of features at each node.

Because of the large imbalance between the number of overlapping and non-overlapping residues in our dataset (see table 1), we obtained a highly unbalanced prediction error between the two classes when using the whole dataset. In order to balance the two class error rates, we applied a down sampling procedure to our dataset. We randomly drew the same number of data points from the majority class as from the minority class to obtain training datasets. This was repeated 1 000 times.

**Table 1:** Confusion matrix for our prediction.

	Observed overlap	Observed non-overlap
Predicted overlap	TP: 672	FP: 1533
Predicted non-overlap	FN: 424	TN: 3186

Here, TP (true positive) and TN (true negative) denote the number of correctly predicted overlap residues and the correctly predicted non-overlap residues respectively. FP (false positive) and FN (false negative) refer to wrong predictions of overlap and non-overlap residues.

## Results and Discussion

This study aims at characterizing the nature of protein residues at overlapping protein-protein and protein-ligand binding interfaces. More precisely, given the three-dimensional structure of such a protein-protein interface, we aimed at developing a method for predicting where small molecule ligands would most likely bind at this interface. In a drug design project targeting a known protein interface, such a method would allow focusing the virtual or experimental screening efforts on ligands with physico-chemical properties that are complementary to the predicted binding patch at the protein interface.

### Statistics of the dataset

**Figure 4:**
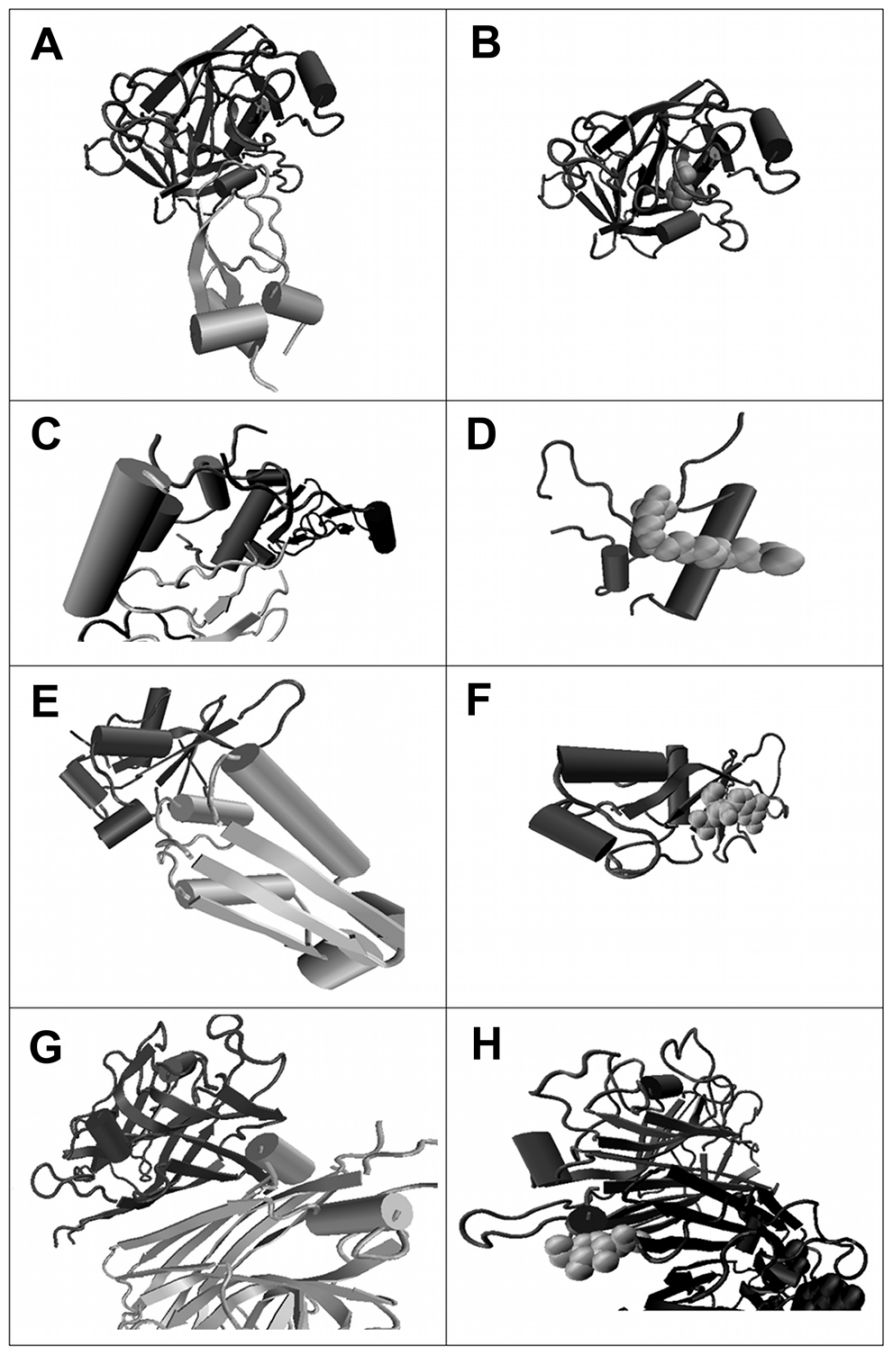
Pairs of protein-protein complexes and the related protein-ligand complexes. (a) and (b) show trypsin bound to the native protein inhibitor Bovine Pancreatic Trypsin Inhibitor (BPTI) (a, PDB code 1BZX E:I) or to the small-molecule ligand benzamidine (b, 1MBQ A:BEN). Here, the identifiers denote the chains and ligands used. The chain identifiers of the reference proteins are marked in bold. (c) and (d) show insulin growth factor protein bound to an IGF-binding protein (c, 2DSQ I:G) or to a detergent molecule (d, 1GZR B:C15). (e) and (f) show barnase bound to the protein inhibitor barstar (e, 1X1U A:D) or to its natural ligand RNA, in this case the tri-nucleotide GMP (f, 1GOY A:3GP). (g) and (h) display a sugar binding protein and its natural ligand N-acetylglucosamine (h, 1DBN A:NAG) or when it forms a quaternary homomeric structure (g, 2DVG C:B).

As explained in the Materials and Methods section, we derived a dataset of 175 tuples (P_i1_, P_i2_) ∶ (P_i3_, L_j_), where P_i1_, P_i2_ and P_i3_ are three proteins and L_j_ is a small molecule ligand, P_i1_ and P_i3_ share at least 40% sequence identity, and the aligned positions in the binding interfaces of P_i1_ – P_i2_ and P_i3_ – L_j_ have at least two residues in common. Fig. 4 shows four representative examples of such tuples. A list of all 175 pairs is available in supplementary table S1. In the PP:PL complex pair 1MBQ-1BZX, for example, the digestion enyzme trypsin is either bound to the small-molecule ligand benzamidine in the PL complex (Fig. 4b) or to the native protein inhibitor Bovine Pancreatic Trypsin Inhibitor (BPTI) in the PP complex (Fig. 4a). The pair 1GZR-2DSQ refers to interactions of the insulin growth factor protein with an IGF-binding protein (PP, Fig. 4c) or with a detergent molecule (PL, Fig. 4d). Here, the PP structure contains further protein chains that are colored grey in the figure, forming a multimeric complex with four chains. The PP:PL pair 1GOY-1X1U involves the well-known barnase-barstar system. Here, the functional roles of ligand and partner protein are reversed. In the PL interaction, the ribonuclease barnase is bound to its natural ligand, the tri-nucleotide GMP (Fig. 4f), whereas the protein binding partner barstar acts as inhibitor in the PP complex (Fig. 4e). In the automatic derivation of the dataset the biological function of the ligand was not considered because we assume that, irrespective of the functional relationship, already the mere existence of a binding site for a small ligand provides valuable information for finding potential drug targets.

The last example in Fig. 4, 1DBN-2DVG, illustrates another class of PP:PL pairs in our dataset. In this case, the PL pair is formed by a sugar binding protein and its natural ligand N-acetylglucosamine (Fig. 4h). Additionally, the PL complex contains a second chain of the sugar binding protein forming a homodimer and the ligand is integrated into the binding interface of the homodimer. The corresponding PP complex was extracted from a quaternary homomeric structure (Fig. 4g). Both PDB files thus contain an equivalent protein-protein complex. The collision filter used in our workflow also picked up this kind of pairs because the ligand is so tightly embedded in the interface area (see methods section). Our final dataset contains about 20 of such cases. Although we could have manually removed these structures, such data arguably also provides valid information for the derivation of the desired prediction approach so that we kept these structures.

**Figure 5:**
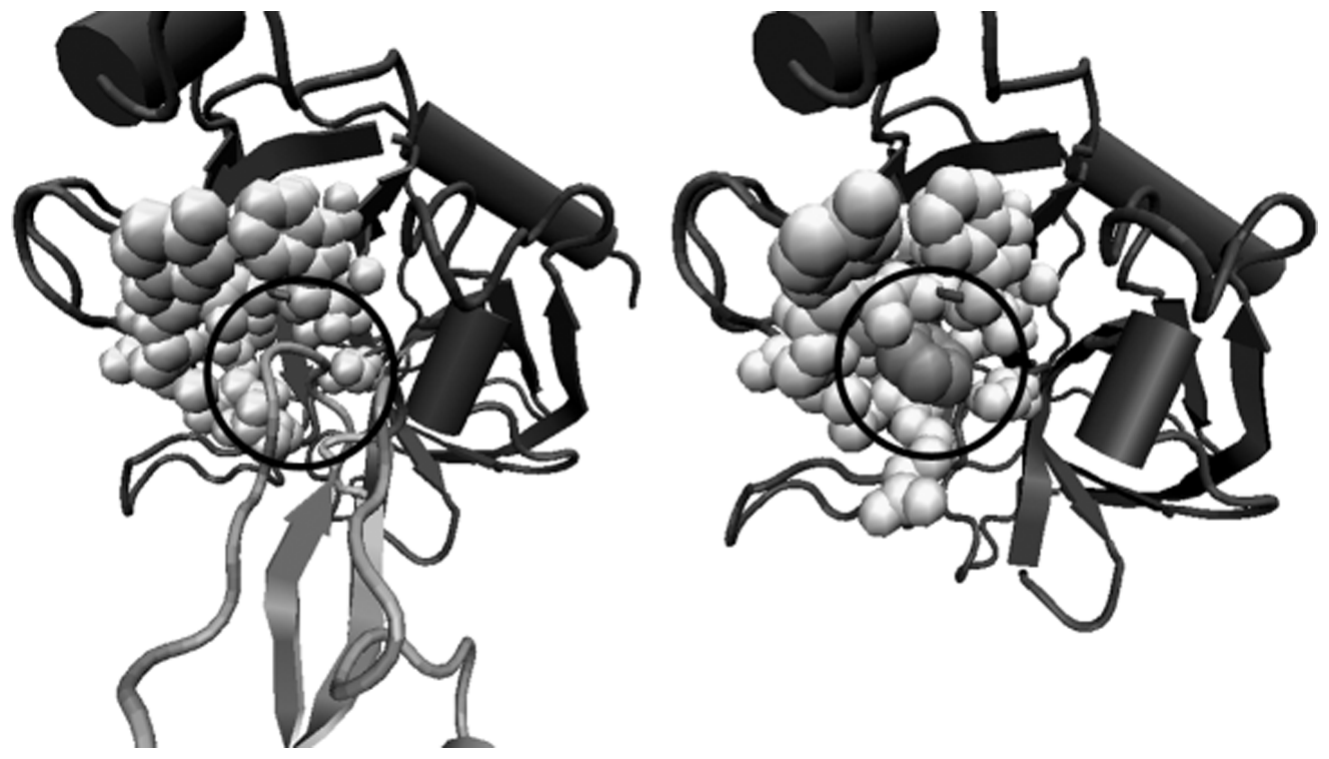
PP:PL pair 1BZX E:I (left) and 1MBQ A:BEN (right). The reference proteins are marked in dark grey, the ligand and the binding protein partner in light grey. Additionally, the overlapping residues on the surface of the reference proteins are marked as bright spheres. The corresponding regions are circled.

The geometric relation between a PP:PL pair is illustrated in more detail in Fig. 5 on the example of the well-known trypsin-benzamidine complex introduced before. The left picture shows how native BPTI binds to trypsin and blocks access to its active site. The right picture shows how a small benzamidine molecule binds into the active-site cavity at the trypsin-BPTI interface and thereby blocks the binding interface for trypsin inhibitor proteins.

The paired PP and PL interfaces in our dataset involve on average 35.5±24.0 P_i1_ residues (PP) and 8.7±6.1 P_i3_ residues (PL). On average, only 25% of the residues in a PP interface participate in the overlapping region whereas 79% of the residues of the corresponding PL interface belong to the overlap region. In fact, 64 PL interfaces out of the 175 PP:PL pairs (37%) were fully covered by the overlapping area compared to none of the PP interfaces.

**Figure 6:**
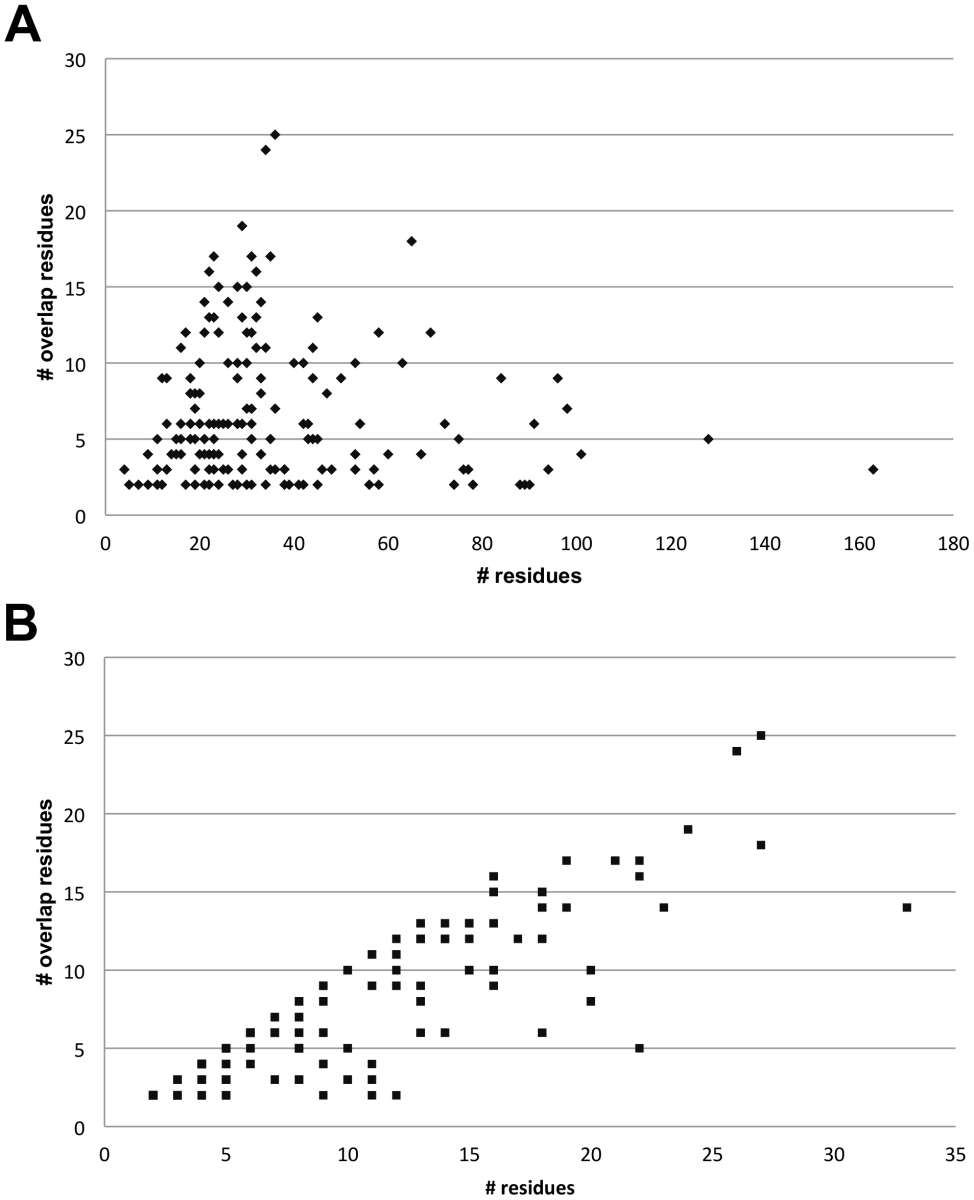
Interface statistics. Shown is the total number of interface residues against (a) the number of overlap residues for PP interfaces and (b) the number of overlap residues for PL interfaces.

Concerning the size of the ligand, we found somehow unexpectedly that the number of ligand atoms was not related to the size of the ligand binding interface (Pearson correlation coefficient equals −0.07). On the other hand, Fig. 6 plots the numbers of overlap residues relative to the total number of interface residues for PP and PL complexes, respectively. For the PP interfaces, no relation is found between the total number of interface residues and the number of overlap residues (−0.06 Pearson). In contrast, the number of overlap residues for PL interfaces is strongly correlated with the total number of interface residues (0.86 Pearson).

### Distribution of interface features

For deriving the statistical classifier, we needed to identify features of the residues at binding interfaces that display different distributions for residues in the overlapping part of PP:PL interfaces and for non-overlapping residues. For this, we tested several structural and evolutionary features of the interfaces that were discussed previously in the literature.

**Figure 7:**
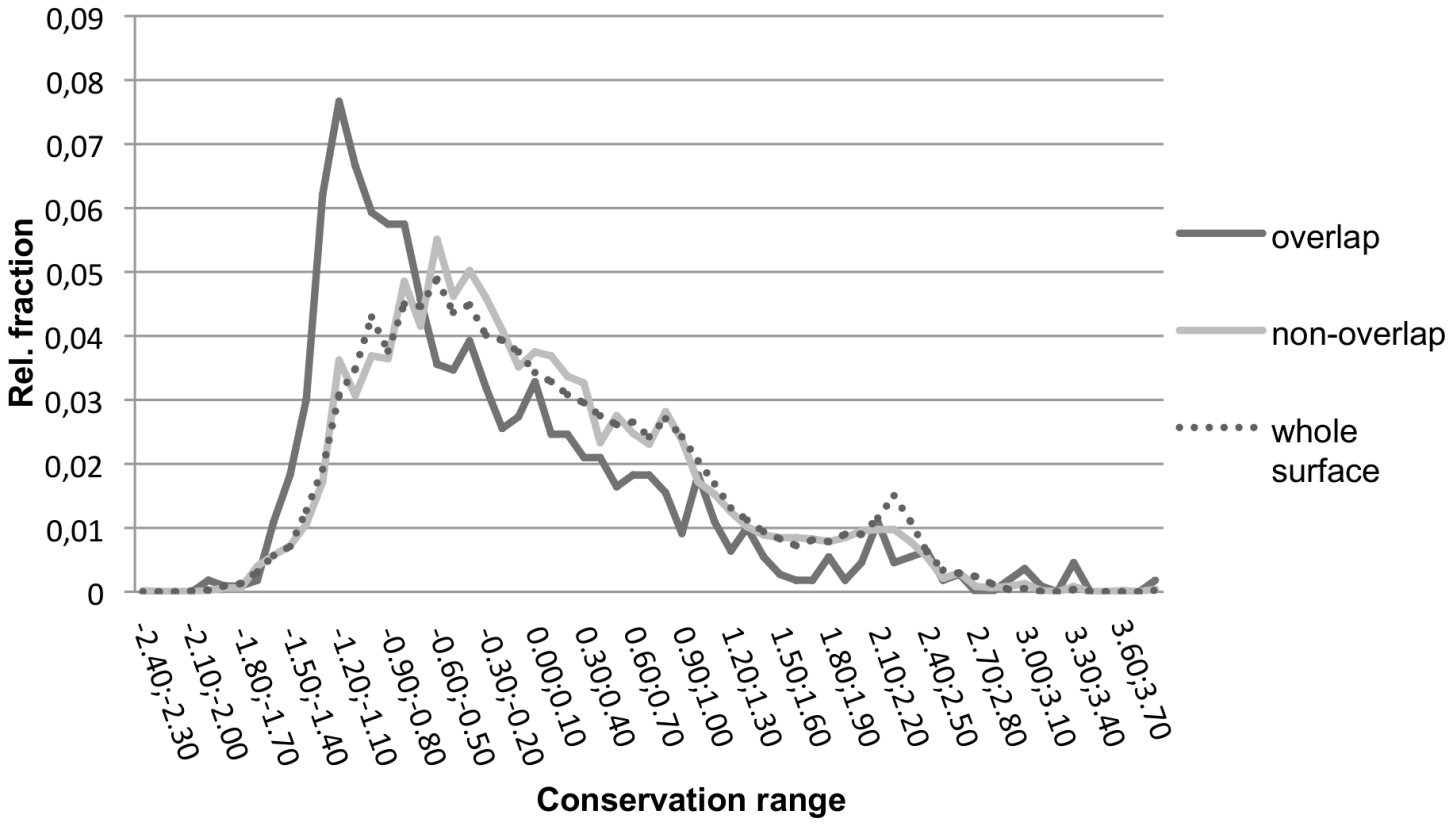
Distribution of conservation ranges obtained from the Consurf webserver. Conservation scores are shown separately for overlap (black line) and non-overlap (grey line) residues for PP interfaces. As a reference, the conservation of all residues on the protein surface (dotted line) is plotted. Negative values indicate residues that are more conserved. Using the Welch t-test the two classes showed a statistically significant difference (p-value<2.2e-16).

Due to their importance for the stability of the complex, residues at binding interfaces generally represent essential functional areas and thus may be potentially preserved during evolution. However, it is yet an unsettled issue whether binding interfaces are generally more conserved than the rest of the protein surface [38]–[40]. Interestingly, we found that residues in the overlapping part of the protein-protein and protein-ligand interfaces are more conserved than non-overlapping residues, see Fig. 7. This finding differs from those reported by Davis and Sali who observed a lower conservation for overlap residues in comparison with non-overlap residues [24]. Davis and Sali first removed redundant binding sites that shared more than 90% of their corresponding alignment positions by grouping them together and choosing randomly a representative member. This procedure is certainly different from the one used here. Moreover, they quantified the conservation of each alignment position using either the number of residue types that occurred at the position or a Shannon entropy-like score. Both approaches assigned lower conservation to ‘bi-functional’ residues. In contrast, the rate4 site algorithm[41] used by Consurf-DB assigns the conservation score at a site as the evolutionary rate of this site by considering the stochastic processes underlying sequence evolution within protein families and the phylogenetic tree of the proteins in the family. Thus, also the way of computing conservation differs between our approach and that of Davis and Sali.

Also Koes and Camacho found a slight preference for predicted SMISPs to be less conserved than the rest of the interface. [26] These SMISP residues are defined as residues of the partner protein P_i2_ that overlap with a high-affinity ligand in a corresponding protein:ligand structure. Obviously, the conservation of P_i2_ residues may differ from that of P_i1_ residues that are considered here. As a reference point, we also computed the conservation of the overall protein surfaces in our data set (see Fig. 7). This distribution is highly similar to that for the non-overlap residues at the interfaces.

Secondly, as mentioned in the introduction, there often exists a small subset of residues, termed hot-spots, that has a larger contribution to the binding affinity than the remaining amino acids of the binding interface. We found that predicted hot spots are underrepresented in the non-overlap regions of protein-protein interfaces (37% hot-spot, 63% non hot-spot) whereas they are equally abundant as non-hot spot residues in the overlap regions (48% hot-spot, 52% non hot-spot). This observation can be interpreted that due to their relatively small size, ligands are engaged in a relatively larger number of contacts with energetically important residues than corresponding protein binding partners.

**Figure 8:**
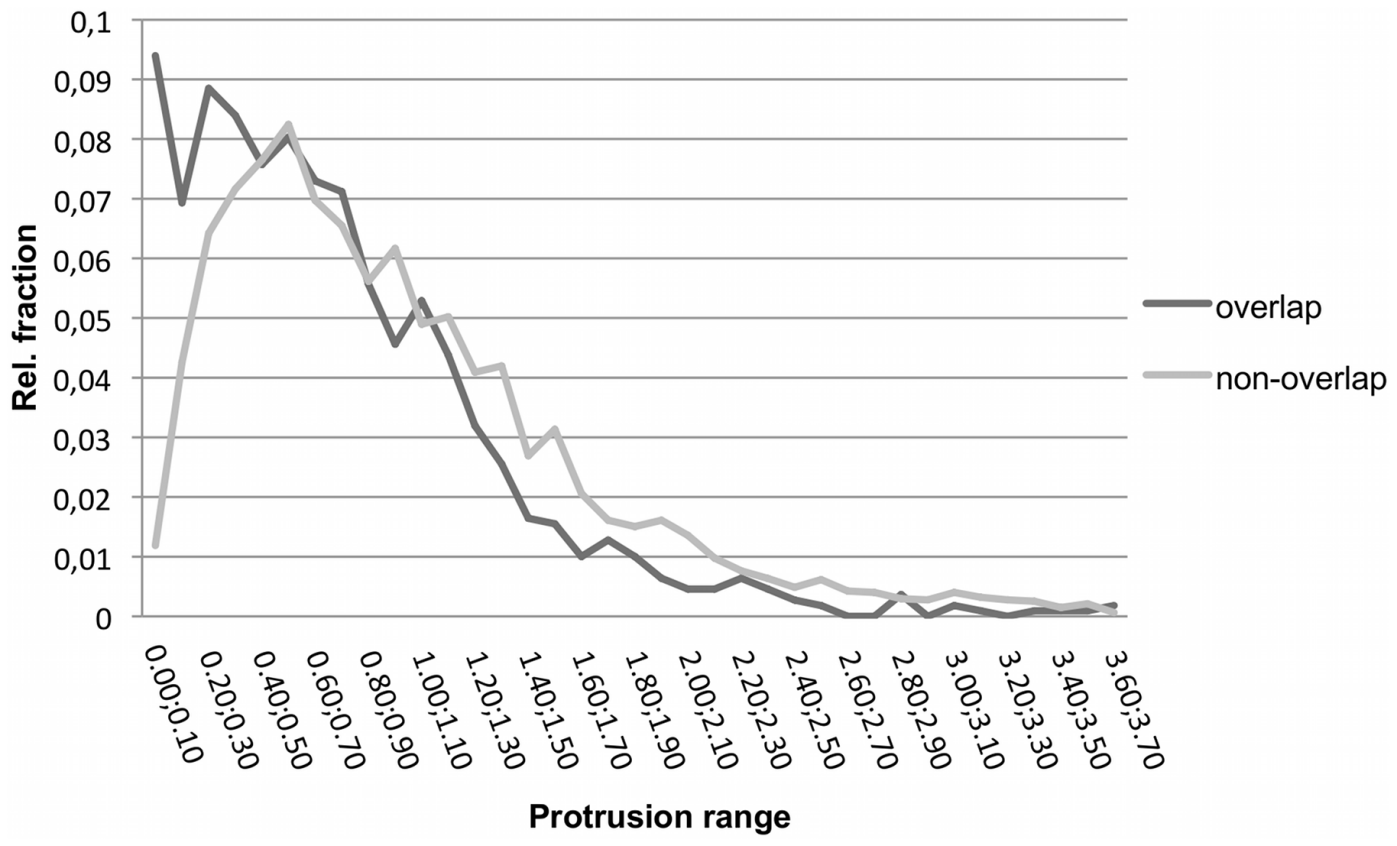
Binned distribution of the protrusion index for overlap and non-overlap residues of PP interactions. Values close to zero indicate buried residues. Using the Welch t-test the two classes showed a statistically significant difference (p-value < 2.2e-16).

The protrusion index, computed on the basis of the P_i1_–P_i2_ complex structure, also shows clearly distinct distributions for overlapping and non-overlapping residues, see Fig. 8. In agreement with previous results [18], [42], the comparatively smaller values found for overlapping residues reflect that they tend to be located in concave structural clefts at the binding interfaces. In contrast, non-overlapping residues tend to have larger protrusion values indicative of exposed locations.

**Figure 9:**
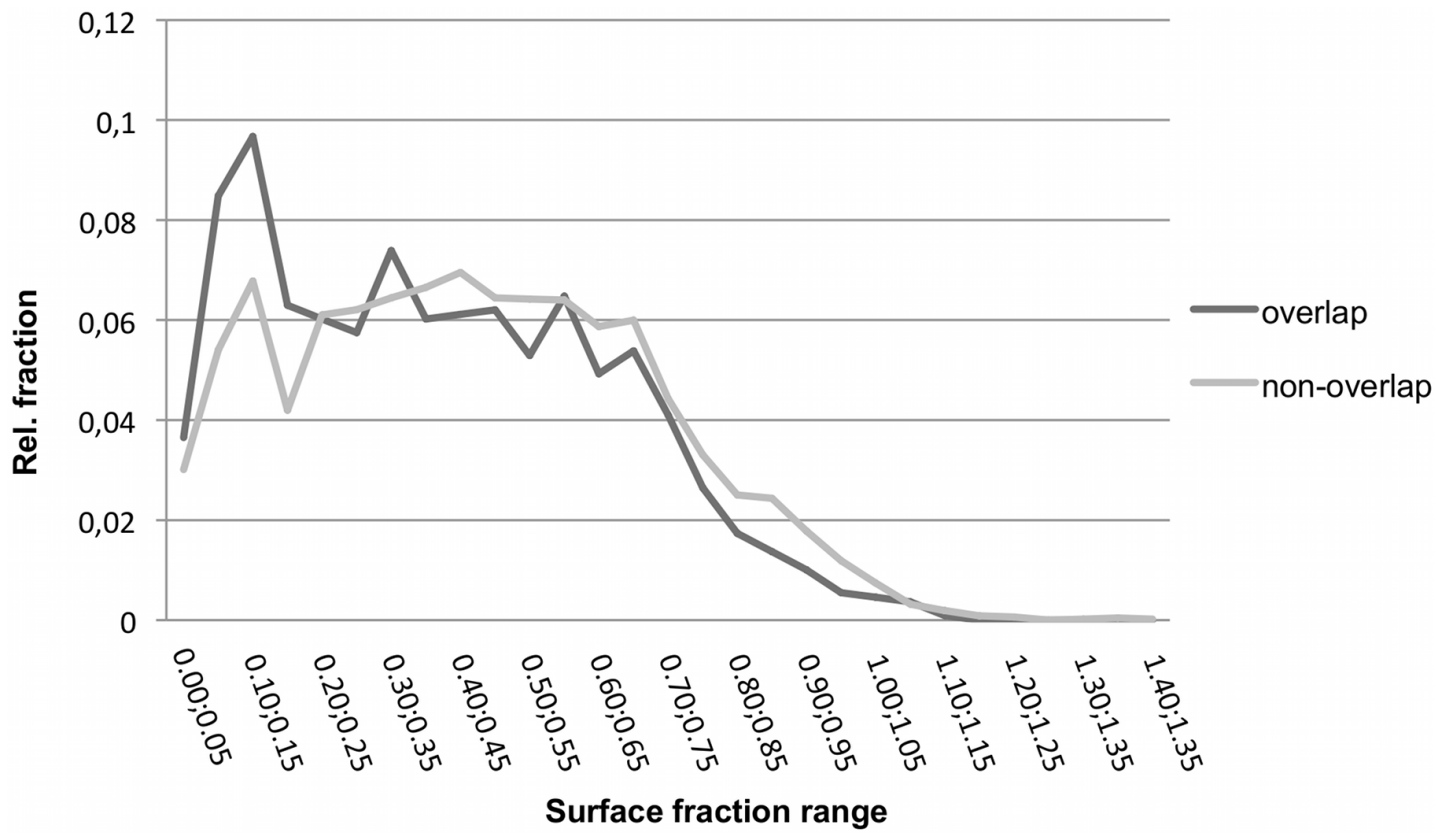
Distribution of surface fractions for overlap and non-overlap residues for PP interfaces. Using the Welch t-test the two classes showed a statistically significant difference (p-value = 6.373e-11).

Fig. 9 shows the relative surface fractions for overlap and non-overlap residues. Residues in the overlapping region have a statistically significant tendency for low surface accessibility. This matches their higher preference of being located in pocket regions as indicated by the protrusion index just discussed. Koes and Camacho found on their dataset that the difference in SASA was the most informative feature for their statistical classifier [26].

The contact density of a residue, that is the average contact density of its solvent accessible heavy atoms, was computed following the work of Illingworth et al.[32]. They reported that residues within ligand binding sites tend to have a higher frequency of contact neighbors than surface residues in general and the density values for hot-spot residues were significantly higher than for non hot-spot residues [16]. Also in our case, the overlap residues show a 0.97 higher contact density than the non-overlap residues in the dataset. The two density distributions (not shown) were statistically significantly different (p-value<1.812e-10) using the Welch t-test.

### Performance of statistical classifier

**Figure 10:**
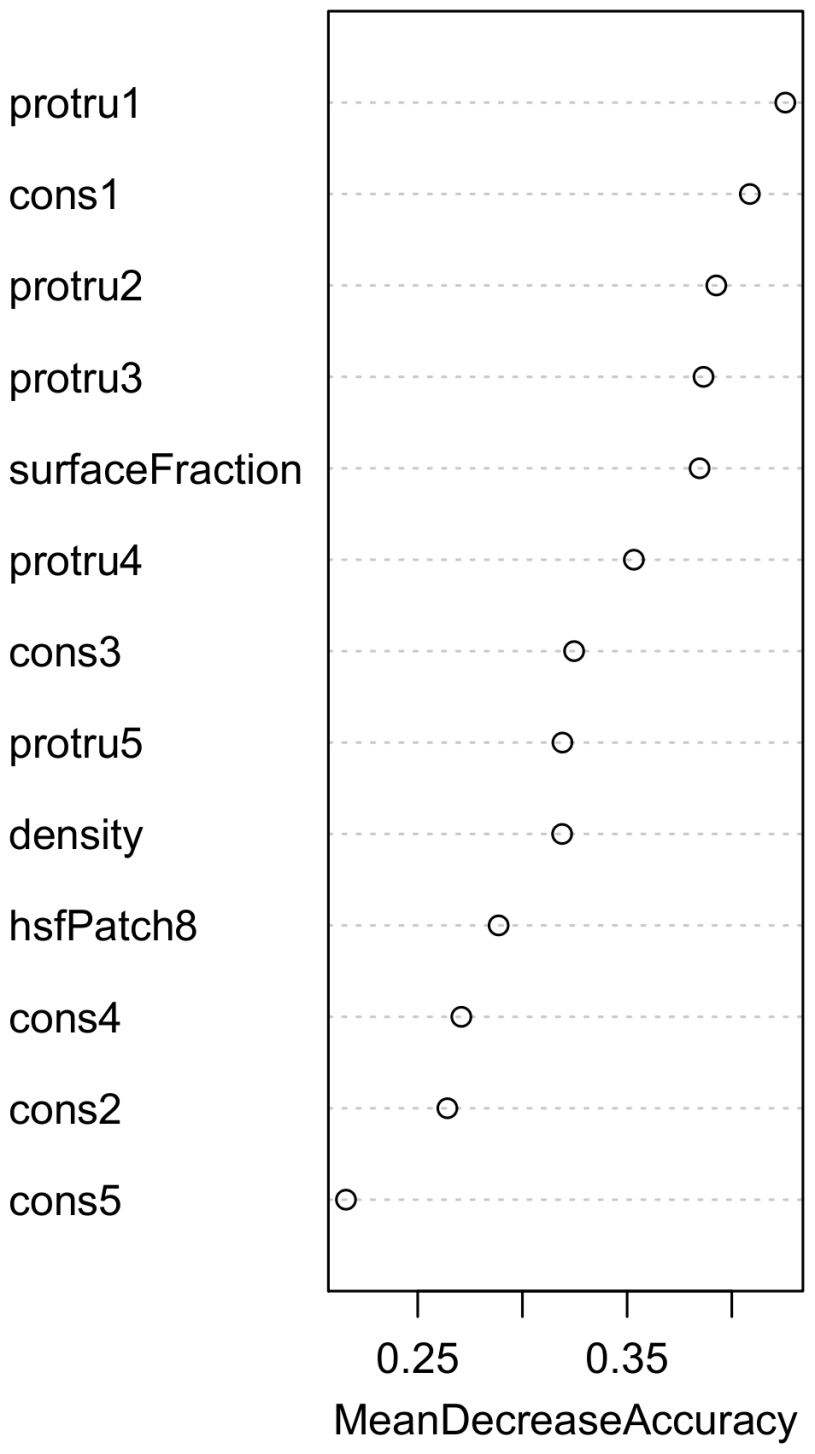
Significance of interface features for predicting overlap residues. MeanDecreaseAccuracy reflects the suitability of a feature as a reliable predictor. Precisely, it reflects how much the average prediction accuracy decreased when randomly shuffled values were used for a particular feature in the testing phase. In the diagram, this quality measure decreases from top to bottom. Cons#n refers to the conservation score of the n-th nearest surface residue starting from the central residue (*n* = 1∶ central residue itself). Analogously, protru#n refers to the protrusion value of the n-th nearest residue starting from the central residue (*n* = 1∶ central residue itself). Density and surfaceFraction describe the contact density and the surface fraction of the central residue. HsfPatch8 denotes the relative frequency of predicted hot-spots in a surface patch of size 8.

Based on these five features (conservation score, hot spots, protrusion index, surface fraction, contact density) we constructed a statistical classifier using random forests [36]. In order to improve our predictions we used the concept of surface patches as described in the Materials and Methods section to include the properties of neighboring residues for the prediction of a central residue. Our final classifier utilizes the surface fraction and contact density of the central residue, the protrusion index and conservation scores of the five nearest residues (including the central residue) and the relative frequency of predicted hot-spots in a surface patch of size 8. Fig. 10 shows the importance of individual features for the construction of the decision tree. Shown is the mean decrease of the accuracy of the predictor if randomly shuffled values were used for a particular feature in the testing phase. For example, using shuffled values for the protrusion of the central residue (protru1) affects the prediction accuracy by more than 40% relative to the performance with the correct values for this feature. In summary, the protrusion index, the conservation score and surface fraction turned out as promising features for distinguishing overlap (O) and non-overlap (N) residues. The accuracy of single residues according to this O/N classification was higher (67%) than the random value (50%) for a binary decision between two classes of the same size (see methods). Table 1 provides an overview of the assigned classifications.

**Figure 11:**
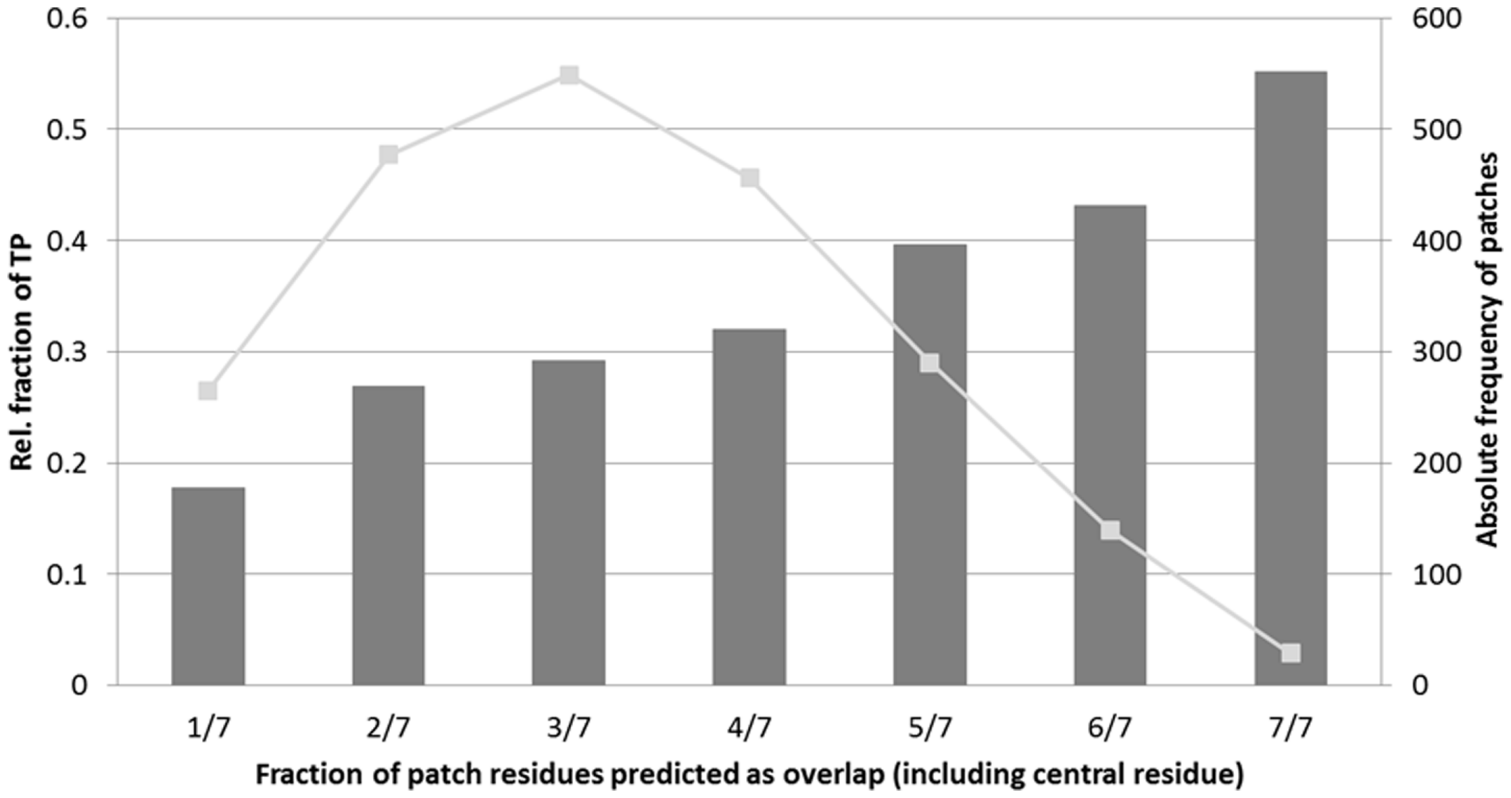
Surface-patch based predictions. The dark grey bars indicate the fraction of positive classifications for 7-residues-patches with increasing proportion of predicted overlap residues (1 out of 7 to 7 out of 7 predicted overlap residues). The light grey line represents the absolute frequency for every patch, e.g. there are about 300 patches for which 5 out of 7 residues in the patch were predicted as ‘overlap’.

Subsequently, we tested whether the values of neighboring individual residues can be used in a ‘patch’ analysis to boost the accuracy of the prediction for the central residue of this patch. For this, we measured the coherence of overlapping regions at PP interfaces. To this end, for every PP interface, the Euclidian distances between the heavy atoms of all residues were calculated and assigned to clusters. A cluster consists of a set of residues in which every residue has at least one neighboring residue in the same set within a distance of 5 Å. We found that 82% of the overlapping regions contained only one cluster, 13% contained two clusters, and the remaining 5% contained three clusters. This observation indicates that, as expected, overlap residues are not spread out over the interface but are located close to each other. Thus, we applied surface patches as described in the methods section as a further means for characterizing this class of residues. We tested patches of *n* = 5 to 8 residues around all central residues that were predicted as overlap residue (‘O’). Consequently, the patches may contain O/(O+N) fractions of 1/*n* to *n*/*n* ‘O’ residues. Fig. 11 shows the frequency of 7-residue patches with different O/(O+N) ratios and the achieved coverage. The respective statistics for the other patch sizes are available as supporting information (Figs. S2–S5).

Fig. 11 shows that a large frequency of predicted overlap residues in a patch increases the probability of true positive hits and thus reduces the risk of misclassifications. Patches appear thus as a suitable method to improve the accuracy of the prediction. Examples for structures in which central residues of patches with O/(O+N) ratio of 1 actually predict true overlap residues are 1Y48 E:I, 1OO9 A:B and 1FAK H:I. All of these pairs describe a complex between a protein and its protein inhibitor. In the corresponding PL complexes, the inhibiting protein is replaced by a small molecule taking over the same role. Among the few incorrect predictions with a O/(O+N) ratio of 1.0, the pair 2OL4 B:JPN and 1NHG B:D turned out to be a special case. Examining the PP structure revealed that the protein chains B and D were apparently split into two chains making it appear as a complex because no electron density could be detected for the intervening residue stretch. However, the protein naturally occurs as a single chain monomer. The prediction process thus recognized four patches with O/(O+N) ratios of 1 that were all wrong. Leaving out this pair improves the fraction of true positives for the maximum overlap up to 67%. For the 175 PP:PL tuples considered, 99 (or about 60%) contained patches with 0.86 or 1.0 ratio.

### Practical case study ‘apoptosis’

**Figure 12:**
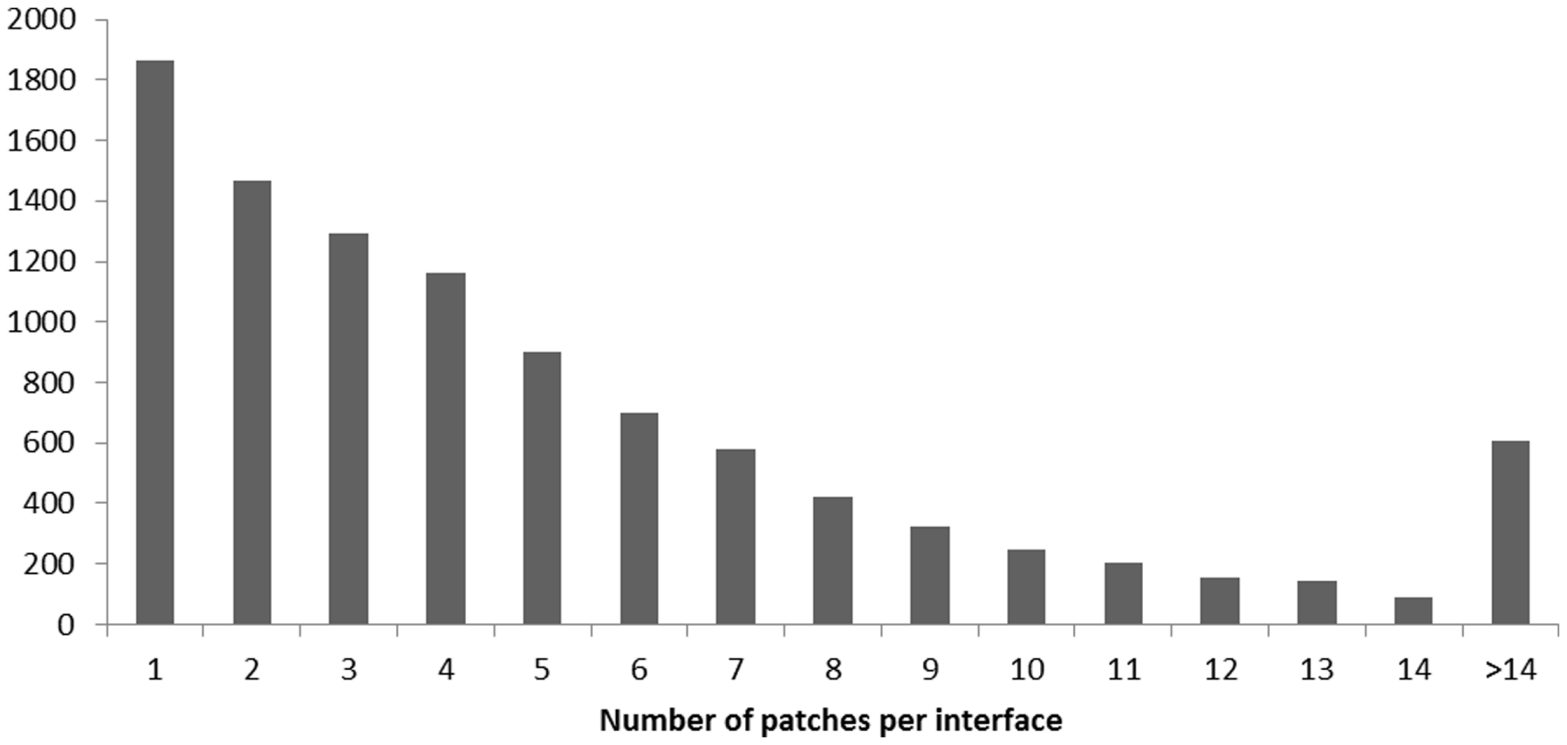
Distribution of the number of 7-residue surface patches per protein-protein binding interface. The central residue must be predicted as ‘overlap’ and at least 4 of the 6 remaining residues must be predicted as ‘overlap’ as well.

We then applied the prediction algorithm to the entire dataset of 48 440 interfaces that is currently stored in our ABCsquare database. To this end, we defined one chain for every interface as reference protein. We then generated surface patches of size 7 for the interface residues of the reference protein and focused on patches where the center residue is predicted as overlap and the total number of overlap residues is at least 5. Altogether we found 54 809 such patches belonging to 10 156 interfaces. (The entire list is provided in supporting table S2). Note that these patches all have different central residues but may be overlapping otherwise. The distribution of surface patches is shown in Fig. 12.

For characterizing these interfaces with respect to their biological role, we clustered the corresponding sequences from all reference proteins to exclude redundancy above 30% sequence identity and to get an overview to which sorts of proteins the ones with overlap patches at the interface belong. The non-redundant set of sequences was analyzed regarding the Gene Ontology (GO)-terms for the involved proteins using the ABCsquare database. In tables 2, S3, S4 we list the most frequent GO-terms found for the three GO categories. The p-values reported for the GO terms were computed in R using Fisher's exact test against the background of 48 440 interfaces in the entire dataset and the Benjamini-Hochberg correction [43] was applied to account for multiple testing.

**Table 2:** GO biological process: Term frequencies of non-redundant set and p-values against the entire dataset (Fisher's exact test, Benjamini-Hochberg correction).

Biological process	Frequency	P-value
signal transduction	39	3.212e-01
blood coagulation	32	4.789e-05
positive regulation of cell proliferation	20	1.515e-02
platelet activation	20	1.224e-02
transcription, DNA-dependent	19	2.092e-02
protein phosphorylation	18	1.0
transcription from RNA polymerase II promoter	17	7.883e-01
apoptosis	16	3.573e-03
proteolysis	16	3.558e-06
protein homotetramerization	16	2.805e-01

Complete lists are available in the supporting information (table S5-S7). Naturally, the dominating functional annotations are ‘protein binding’ and ‘protein homodimerization activity’ (see table S3). No preference for a particular cellular compartment was observed (see table S4). Most interesting for medical applications is Table 2 showing the most frequently annotated biological processes.

For a practical case study, we selected all terms from the biological process category containing the expression ‘apoptosis’, see table S8, as these terms may be related to cancer therapy and collected all corresponding interfaces with their predicted overlap residues. This term is significantly enriched (corrected p-value 0.004). Such a list may be a useful source for identifying potential drug targets. Table 3 lists all PDB entries that contain at least three terms related to ‘apoptosis’ and contain predicted overlap patches on one of the protein chains. Interestingly, all but the glyoxalase protein refer to very prominent proteins with central roles in the apoptosis machinery. Based on the reported accuracy for 5/7 patches shown in Fig. 11, the ratio of true positive ligand binding patches among the predicted interface residues should be at least 0.4.

**Table 3:** PDB entries containing ‘overlap’ surface patches that contain at least 3 of the terms related to ‘apoptosis’ listed in Table S8.

PDB+chain	Protein name	Term frequency
1PQ1A	Apoptosis regulator Bcl-X	9
1RE1B	Caspase-3	9
1OLGA	Tumor suppressor P53	7
2C2ZB	Caspase-8 P10 subunit	7
2TNFB	Tumor necrosis factor alpha	5
1DU3D	apo2l/TRAIL	3
1BH5B	Glyoxalase I	3
1PYOD	Caspase-2	3

**Figure 13:**
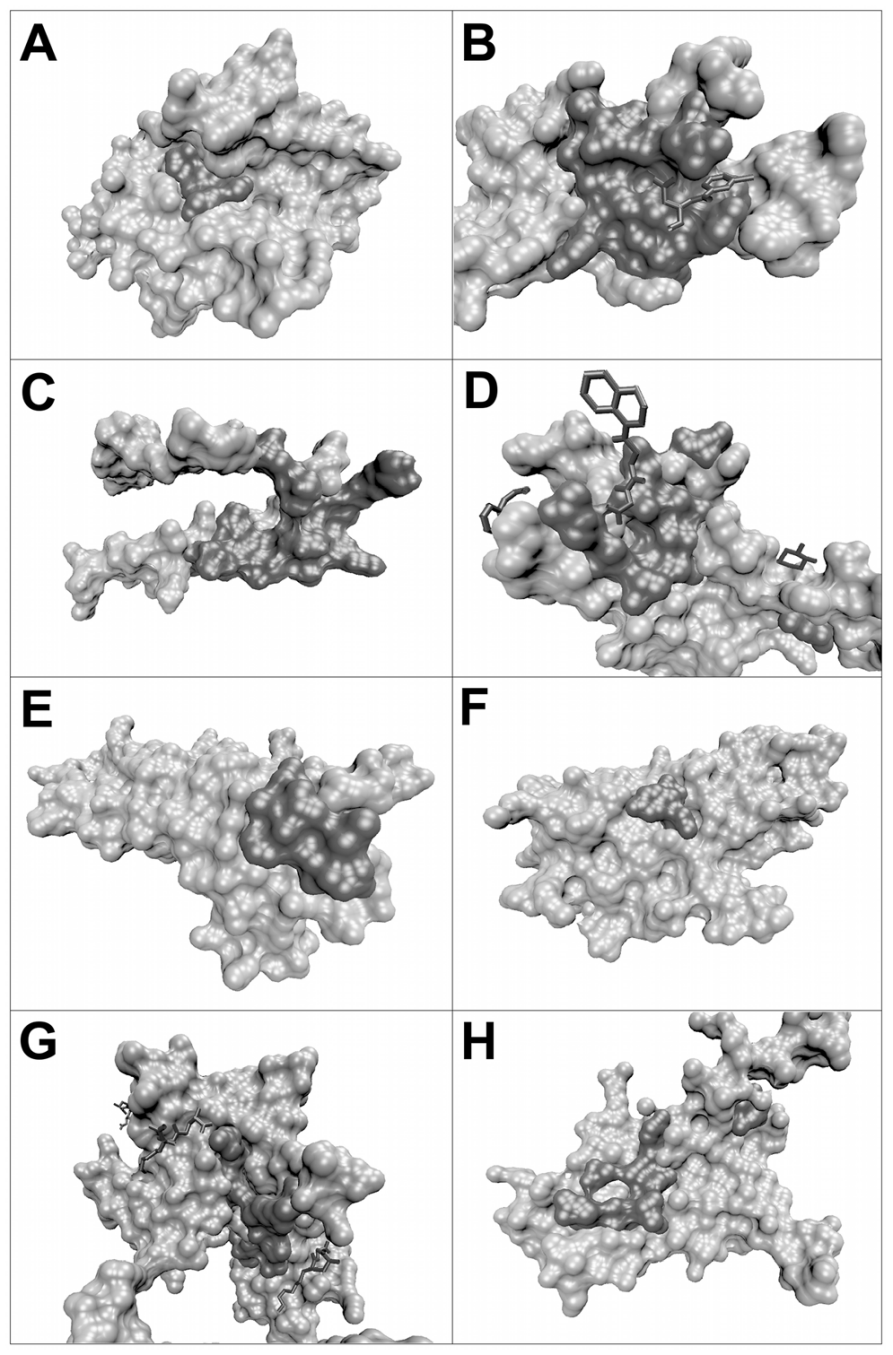
Visualization of complexes related to apoptosis. Predicted overlap residues are colored in dark grey whereas all other surface residues appear in light grey. (a) 1PQ1A, (b) 1RE1B, (c) 1OLGA, (d) 2C2ZB, (e) 2TNFB, (f) 1DU3D, (g) 1BH5B, (h) 1PYOD.

Fig. 13 visualizes the predicted ‘overlap’ surface patches for the eight complexes listed in table 3. Most of the predicted residues form a coherent area on the surface region. Only 1PYOD exhibits a cluster of overlap residues and a single overlap residue. The size of the overlap region ranges from rather small and compact as in 1PQ1A to large as shown in 1OLGA. Interestingly, some of the complexes also contain small ligands. In 1RE1B and 2C2ZB, a small molecule binds to the predicted overlap regions supporting the reliability of the prediction method. In the latter complex the small molecules beyond the overlap area are located in the border region of the protein-protein complex. In 1BH5B, the predicted overlap region does not correspond to the binding site for the small molecules. However, this does of course not exclude the possibility that other ligand molecules may bind to the predicted overlap region.

We emphasize that such predictions do not require the availability of a crystal structure of a given protein-protein complexes. Experimental knowledge about the binding interface, e.g. from chemical shift mapping by NMR or from accessibility measurements is a sufficient basis as input for a prediction by our method.

As a caveat to this analysis we note that this analysis is of course limited by the amount of structural data on protein-protein and protein-ligand complexes currently available. This particularly affects the definition of non-overlap residues. It is clearly possible that these residues could be involved in binding alternative, possibly larger ligands. However, the clearly distinguishable properties of overlap and non-overlap residues derived in this study suggest that there may be only relatively few such cases.

### Concluding remarks

We have presented a new method that analyzes structural and physiochemical features of protein-protein binding interfaces. When given the three-dimensional structure of a protein-protein complex or the structure of a single protein with annotated PP interface, the method is able to identify to which parts of the PP interface small molecules will likely bind. In this regard our method differs from a related method recently presented by Davis [24] that transfers observed ligand positions bound to one protein to the surfaces of related, homologous proteins that may also bind other proteins. We make available predictions for more than 10 000 protein-protein interfaces that can aid researchers to focus their attention on particular portions of these interfaces.

## Supporting Information

Figure S1 **Protein chain surface colored according to protrusion values.** Blue colors were used for atoms that are more exposed to the outside; red refers to atoms that are buried in the structure.
(TIF)

Figure S2 **Maximum overlap patch for patch size 5.**
(TIF)

Figure S3 **Maximum overlap patch for patch size 6.**
(TIF)

Figure S4 **Maximum overlap patch for patch size 7.**
(TIF)

Figure S5 **Maximum overlap patch for patch size 8.**
(TIF)

Table S1 **Dataset of PP:PL pairs.**
(DOC)

Table S2 **Dataset of predicted overlap residues (predictedOverlapResidues.txt).** The first column contains the PDB identifier and the chain name, the second column contains the position number of the residue that represents the central residue of the patch and is predicted as ‘overlap’.
(TXT)

Table S3 **Term frequencies for GO function.**
(DOC)

Table S4 **Term frequencies for GO cellular component.**
(DOC)

Table S5 **GO function terms+frequency that are found in the dataset of predicted overlap residues (predictionGOfunction.txt).**
(TXT)

Table S6 **GO biological process terms+frequency that are found in the dataset of predicted overlap residues (predictionGOprocess.txt).**
(TXT)

Table S7 **GO cellular component terms+frequency that are found in the dataset of predicted overlap residues (predictionGOcellComp.txt).**
(TXT)

Table S8 **Biological process GO terms referring to apoptosis.**
(DOC)
