# Supplementary figures and images for: Predicting where Small Molecules Bind at Protein-Protein Interfaces
Source: PLoS One. 2013 Mar 7;8(3):e58583. doi: 10.1371/journal.pone.0058583 (PMC3591369; doi:10.1371/journal.pone.0058583)

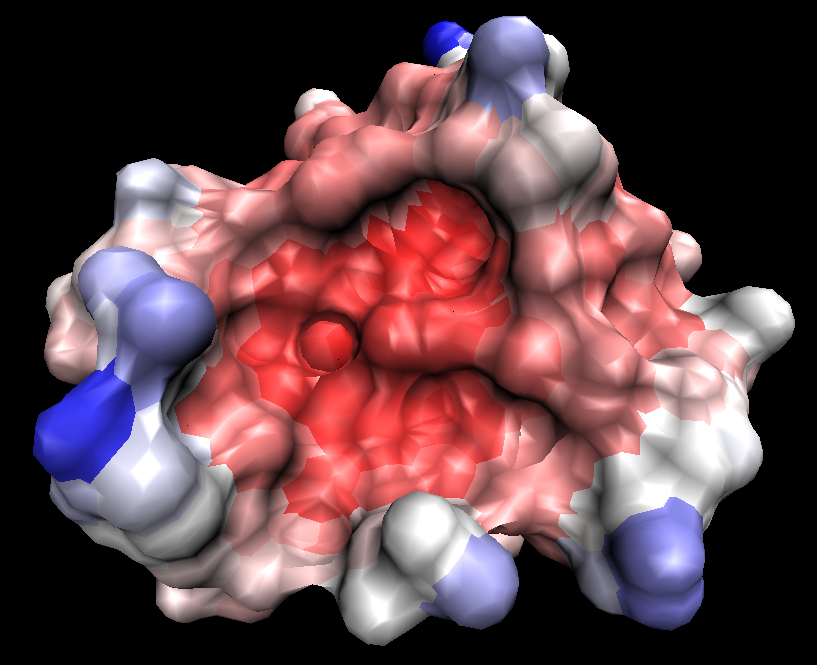

Supplement: Figure S1 — Protein chain surface colored according to protrusion values. Blue colors were used for atoms that are more exposed to the outside; red refers to atoms that are buried in the structure. (TIF) [file pone.0058583.s001.tif]

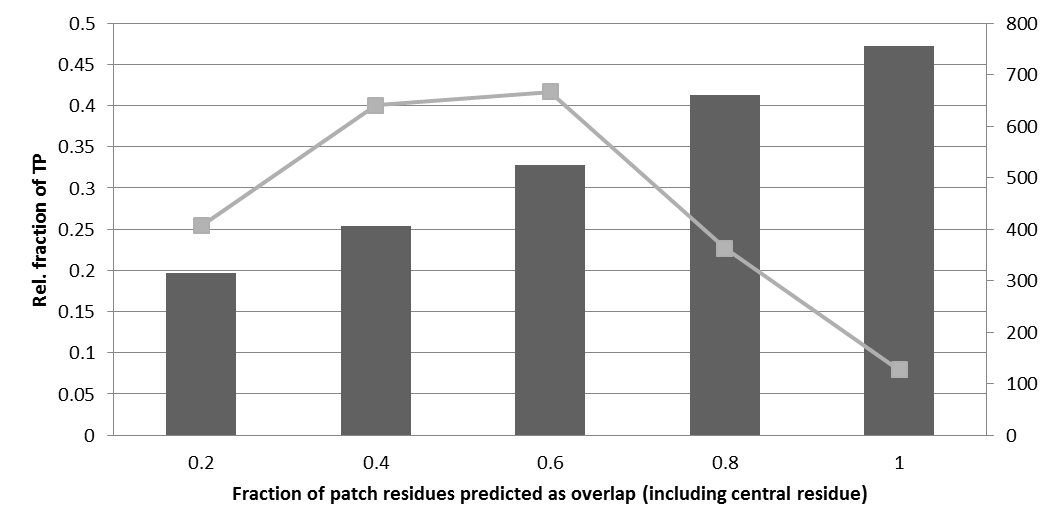

Supplement: Figure S2 — Maximum overlap patch for patch size 5. (TIF) [file pone.0058583.s002.tif]

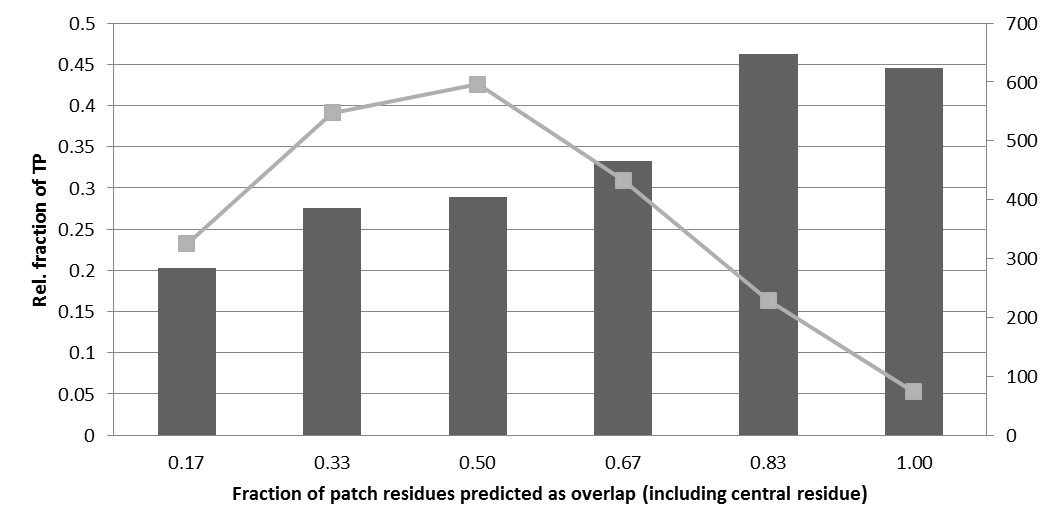

Supplement: Figure S3 — Maximum overlap patch for patch size 6. (TIF) [file pone.0058583.s003.tif]

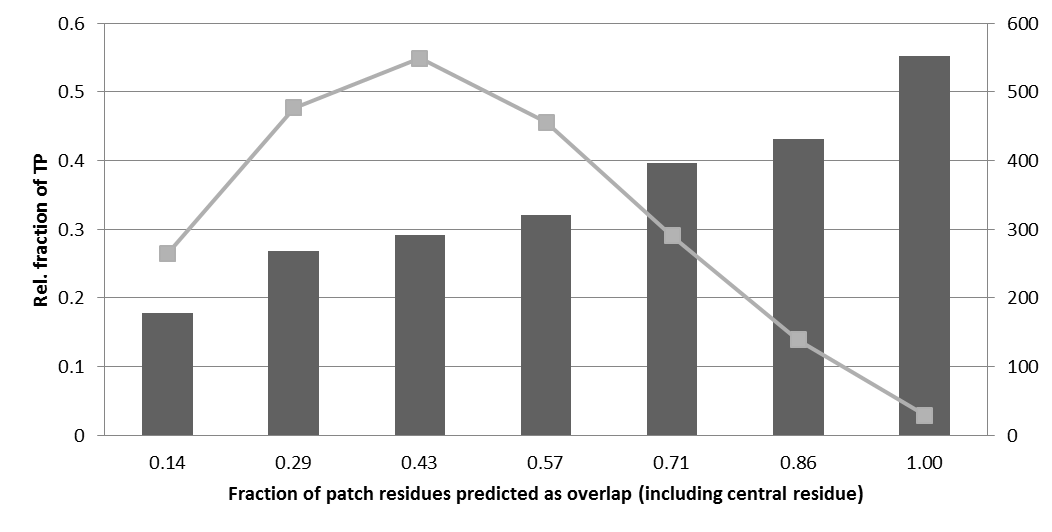

Supplement: Figure S4 — Maximum overlap patch for patch size 7. (TIF) [file pone.0058583.s004.tif]

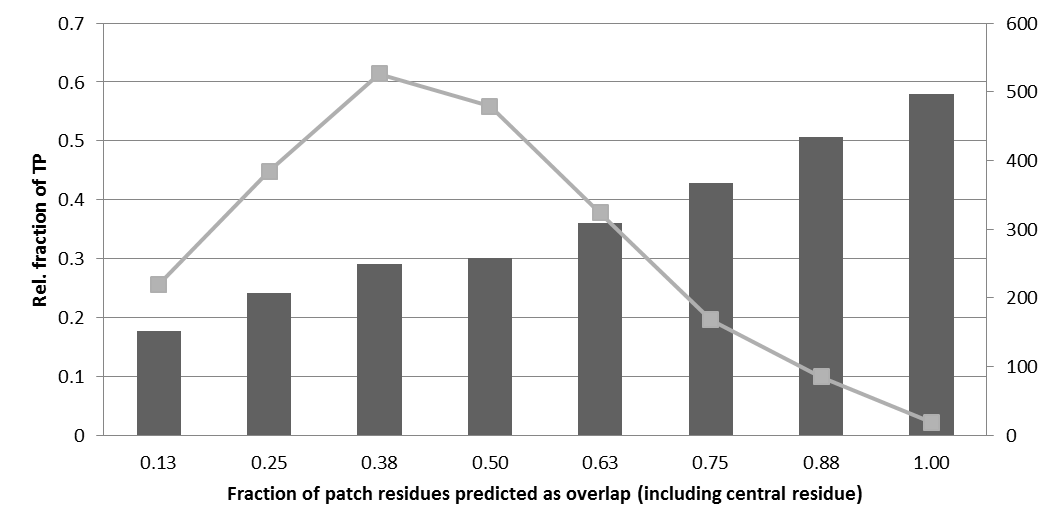

Supplement: Figure S5 — Maximum overlap patch for patch size 8. (TIF) [file pone.0058583.s005.tif]
