# Supplementary material for: Predicting where Small Molecules Bind at Protein-Protein Interfaces
Source: PLoS One. 2013 Mar 7;8(3):e58583. doi: 10.1371/journal.pone.0058583 (PMC3591369; doi:10.1371/journal.pone.0058583)
Supplement: Table S1 — Dataset of PP:PL pairs. (DOC) [file pone.0058583.s006.doc]

PDB Chain Ligand PDB Chain Chain

1MFI B FHC 1GIF B A

1UBH S MPD 1YRQ A H

1LBC A CYZ 1S7Y B A

2HQU C DUP 2OL1 A C

1A05 B IPM 2AYQ B A

1JI5 B MPD 2CHP C D

1C50 A CHI 1YGP A B

1GZR B C15 2DSQ I G

2BUQ A CAQ 3PCD A M

1XKD A NAP 2D4V B A

1V84 A NAG 1KWS B A

1LVW D TYD 1IIN A B

1DHK B NAG 1BJQ C A

1HJ1 A PMB 1U3R A B

1CY2 A TMP 1CYY A B

1AR1 A LDA 2OCC A B

1ANK A AMP 4AKE A B

1USR A SIA 1E8U B A

1Q6Y A MPD 1VGQ B A

1U3T B CCB 1U3W B A

1QIW A DPD 2BE6 A D

1RYD A GLC 1H6A B A

1XR8 A PG4 2NX5 A D

2J9C C ATP 1HWU B A

1CPC A CYC 2J96 B A

1S57 B EPE 1B99 F C

2IPF A TRS 2IPJ B A

35C8 L NOX 15C8 L H

1BCS B CST 1GXS D C

1LOJ C MPD 1N9S C D

1LOJ E MPD 1TH7 L K

2H6Y A MPD 2BTO T A

1H48 C CDI 2AMT C B

1RE2 A NAG 1CKG B A

1DBN B NAG 2DVG C B

2IWZ A 6NA 1W0I B A

9RSA B ADU 1DFJ E I

2H0T A EPE 2G2U A B

1IT6 A CYU 2O8A A I

1YZW C PEG 1ZUX D B

1GOY A 3GP 1X1U A D

2AZ5 B 307 2TNF B A

1Q4J A GTX 1OKT B A

2OIZ A TSR 2AGY B D

1SVL C ADP 2H1L E F

2APX A MLA 2AQ3 G A

2DQV A GAL 1SUV D F

1G4I A MPD 1FX9 B A

1LIN A TFP 2BL0 B A

1X29 B PMG 2AY5 A B

2AY9 B 5PV 1ASL B A

1YZW C PEG 1XMZ B A

2HG8 A MLE 2COG A B

1L9B L HTO 2GMR L M

1MBQ A BEN 1BZX E I

1YRX B D9G 2IYG B A

1G8I A P6G 2I2R H D

2J8C M GGD 1PST M H

1NGP L NPA 1P4I L H

1KYN A KTP 1FI8 A C

1XXS B STE 1PA0 B A

2IW6 A QQ2 1G3N A B

2NY0 A HEZ 2NY7 G H

2FMH A TRS 1VLZ B A

2PL7 B HTG 2GVM B A

1M2Z A BOG 2AAX A B

1A8J L PME 1MCI B A

1OAU J DNF 1A6U H L

1TI1 A D12 2HI7 A B

2C01 X ATP 2BEX C A

1CLS D DEC 1G0A D B

1JTK A THU 1R5T A B

1KJ1 D MAN 1MSA B C

1XEY A GUA 1PMO D C

2CZ5 B CIT 1X1Z A B

3LJR A GGC 2C3N A B

2C97 B MPD 1W29 C D

1L7Z A MYR 1KQM C A

1RFX C PEG 1RH7 C B

1RH7 C P6G 1RFX B C

1GKA A D12 1OBQ B A

2CL0 X TRS 1HE8 B A

1HUR A GDP 1R8Q A E

2FYD A PG4 1HFY A B

1S9Q B CHD 2GPV D B

1UTM A PEA 1SGF G B

1SGC A CST 1SGR E I

1A0J A BEN 1AZZ A C

1O9T B ATP 1P7L A B

1KMH B TTX 1BMF F B

2C97 D MPD 1HQK E D

1TXC A 2AN 1IFV A B

2HA3 A P6G 1C2O D A

2B0U B MPD 1S4Y B A

2JH0 D 701 1E0F E J

1O5D H CR9 1FAK H I

1SUP A PMS 1Y48 E I

1PFK A ADP 6PFK C D

1O6T A MES 1O6S A B

1RHM B NA4 1I3O B E

1EST A TOS 1MCV A I

1HX0 A AC1 1BVN P T

1LOJ A MPD 1I8F C B

1CGY A MAL 1D7F B A

1BIW B S80 1OO9 A B

2P95 A ME5 1P0S H E

1BMQ A MNO 1SC1 A B

4VGC B SRD 1HJA B I

1NIP B ADP 1G21 H G

1MPF A C8E 1OPF B A

1GG6 C APF 1N8O C E

1ICR A NIO 1KQD A B

1HVV A TAR 1JTH D A

1I9B D EPE 1YI5 C H

2FNW A REP 1EZL C D

1WV7 T FUC 1AHW F A

1G5N A SGN 1DM5 B D

1XJI A D10 1BRR A C

2C4L A SIA 1NMA N H

2I17 A CIT 1MI3 B A

1W5F B G2P 1RQ7 B A

1ZOM A 339 1XX9 B D

1RTK A GBS 1DLE A B

1FQ6 A GSC 1DPJ A B

4LIP D CCP 1QGE D E

1SPQ A PEG 7TIM B A

1Y11 A 1PE 2EV4 B A

1ZRK A 367 1ZJD A B

1UX0 A THU 2FR6 C D

1TB6 I MPD 2GD4 I H

2DJH A UM3 2FHZ B A

1RZH H CDL 1EYS H M

1BWO A LPC 1UVC A B

2J6E B MPD 2IWG D E

2OM9 A AJA 2PRG A C

1FLJ A GTT 1G6V A K

1GMR A 2GP 1AY7 A B

2FP7 A NDL 2IJO A I

2G7Y A MO9 1ICF A I

1EKX C PAL 1GQ3 C B

1TR5 A THP 1SND A B

1Q6O B LG6 1XBY B A

1L9H A HTO 1F88 B A

2OL4 B JPN 1NHG B D

2IWZ A 6NA 2GQD B A

1O4H A 772 1A09 A B

1WV0 A BN4 1PYG B A

6RNT A 2AM 1BVI A C

2J7L A XC2 1QPX A B

1IZ2 A SUM 2D26 A B

2FP7 B NDL 2IJO B I

1Y2F A WAI 1F46 B A

2A01 A AC9 1AV1 B A

1I5G A TS5 1O81 B A

2YXJ A N3C 2P1L A B

1U0H B ONM 1AB8 B A

2GJ6 D 3IB 1QRN D E

1BQI A SBA 1STF E I

2UUE B GVC 1JSU B C

2B45 X EPE 1T6G C A

2DCY A TAR 2B42 B A

1F42 A MNB 1F45 A B

2GUI A PEG 2IDO A B

1H1B A 151 1PPF E I

1BG9 A GLC 1AVA A C

1G0T A PEG 1JZD B C

2HXM A 302 1UGH E I

2G2Z A COZ 2CUY B A

1WB8 A PMS 1B06 A B

1BLC A CEM 1OME A B

1EWY C FAD 2PVO D A

2OPY A CO9 1NW9 A B

1ZL0 A TLA 1ZRS B A

1V3V B 5OP 2J3K A B

2GOO C NDG 1NYS A C

**Table S1: Dataset of PP:PL pairs**
