# Supplementary material for: Predicting where Small Molecules Bind at Protein-Protein Interfaces
Source: PLoS One. 2013 Mar 7;8(3):e58583. doi: 10.1371/journal.pone.0058583 (PMC3591369; doi:10.1371/journal.pone.0058583)
Supplement: Table S3 — Term frequencies for GO function. (DOC) [file pone.0058583.s008.doc]

| **Function** | **Frequency** | **P-value** |
| --- | --- | --- |
| protein binding | 341 | 9.455e-02 |
| protein homodimerization activity | 53 | 1.114e-04 |
| identical protein binding | 27 | 3.745e-01 |
| magnesium ion binding | 20 | 3.410e-01 |
| DNA binding | 18 | 4.789e-05 |
| zinc ion binding | 17 | 7.300e-15 |
| sequence-specific DNA binding transcription factor activity | 13 | 2.310e-03 |
| ATP binding | 12 | 2.460e-12 |
| flavin adenine dinucleotide binding | 12 | 1.756e-21 |
| transcription activator activity | 12 | 5.151e-01 |

**Table S3:** GO function: Term frequencies of non-redunant set and p-values against the entire dataset (Fisher’s exact test, Benjamini-Hochberg correction).
