# Supplementary material for: Predicting where Small Molecules Bind at Protein-Protein Interfaces
Source: PLoS One. 2013 Mar 7;8(3):e58583. doi: 10.1371/journal.pone.0058583 (PMC3591369; doi:10.1371/journal.pone.0058583)
Supplement: Table S4 — Term frequencies for GO cellular component. (DOC) [file pone.0058583.s009.doc]

| **Cellular component** | **Frequency** | **P-values** |
| --- | --- | --- |
| cytoplasm | 129 | 6.954e-18 |
| cytosol | 121 | 7.226e-12 |
| nucleus | 96 | 7.221e-02 |
| plasma membrane | 69 | 2.961e-02 |
| mitochondrion | 61 | 6.318e-01 |
| extracellular region | 49 | 1.664e-10 |
| nucleoplasm | 36 | 1.382e-26 |
| extracellular space | 33 | 1.722e-3 |
| nucleolus | 26 | 7.488e-02 |
| membrane | 24 | 3.724e-07 |

**Table S4:** GO cellular component: Term frequencies of non-redunant set and p-values against the entire dataset (Fisher’s exact test, Benjamini-Hochberg correction).
