# Supplementary material for: Predicting where Small Molecules Bind at Protein-Protein Interfaces
Source: PLoS One. 2013 Mar 7;8(3):e58583. doi: 10.1371/journal.pone.0058583 (PMC3591369; doi:10.1371/journal.pone.0058583)
Supplement: Table S8 — Biological process GO terms referring to apoptosis. (DOC) [file pone.0058583.s013.doc]

| **Biological process GO terms related to apoptosis** | **Frequency** | **P-value** |
| --- | --- | --- |
| apoptosis | 16 | 3.573e-03 |
| anti-apoptosis | 12 | 4.579e-04 |
| induction of apoptosis | 8 | 1.561e-04 |
| negative regulation of apoptosis | 8 | 7.480e-01 |
| induction of apoptosis by extracellular signals | 7 | 3.431e-02 |
| regulation of apoptosis | 5 | 7.003e-02 |
| cellular component disassembly involved in apoptosis | 5 | 3.363e-06 |
| induction of apoptosis by intracellular signals | 4 | 2.369e-05 |
| negative regulation of neuron apoptosis | 4 | 4.540e-01 |
| positive regulation of neuron apoptosis | 2 | 3.431e-02 |
| DNA damage response, signal transduction by p53 class mediator resulting in induction of apoptosis | 2 | 7.480e-01 |
| positive regulation of anti-apoptosis | 2 | 1.0 |
| negative regulation of B cell apoptosis | 1 | 1.0 |
| transformed cell apoptosis | 1 | 1.222e-02 |
| negative regulation of smooth muscle cell apoptosis | 1 | 4.590e-01 |
| induction of apoptosis via death domain receptors | 1 | 1.476e-06 |
| positive regulation of thymocyte apoptosis | 1 | 8.756e-01 |

**Table S8:** Biological process GO terms referring to apoptosis with p-values against the entire dataset (Fisher’s exact test, Benjamini-Hochberg correction).
